# Transitional care for older persons with need of geriatric rehabilitation nursing interventions

**DOI:** 10.1186/s12912-024-02050-4

**Published:** 2024-06-04

**Authors:** Rogério Ferreira, Ana Rita Pedrosa, Neuza Reis, Luís Sousa, Célia Nicolau, Bruno Ferreira, Belmiro Rocha, Cristina Lavareda Baixinho

**Affiliations:** 1 Polytechnic Institute of Beja, Higher School of Health, Rua Pedro Soares, 7800-295 Beja, Portugal; 2 Comprehensive Health Research Centre (CHRC), 7000-811 Évora, Portugal; 3 Nursing Research, Innovation and Development Centre of Lisbon (CIDNUR), 1900-160 Lisbon, Portugal; 4 Higher School of Atlantic Health, 2730-036 Barcarena, Oeiras, Portugal; 5 Alentejo Coastal Hospital (HLA), Alentejo Coastal Local Health Unit (ULSLA), 7540-230 Santiago do Cacém, Portugal; 6 Polytechnic Institute of Setúbal, Higher School of Health, 2910-761 Setúbal, Portugal; 7 Associação Portuguesa de Enfermeiros de Reabilitação (APER), 4500-627 Silvalde, Portugal; 8 Center for Innovative Care and Health Technology (ciTechcare), 2410-541 Leiria, Portugal

**Keywords:** Nursing, Rehabilitation, Transitional care, Continuity of Care, Functionality

## Abstract

**Background:**

The literature review notes that people in need of care from Rehabilitation Programs do not always see their continuity ensured.

**Objective:**

This study aim to analyze the perspective of Specialists Nurse in Rehabilitation Nursing in relation to the organization and specialized intervention of transitional care for older people in need of rehabilitation programs.

**Methods:**

This is a qualitative study within the interpretivist paradigm. A focus group with 8 nurses and 13 interviews with Portuguese nurses were carried out between April 2022 and February 2023. Content analysis was carried out.

**Results:**

The triangulation of the data made it possible to identify 3 categories: Coordination of a transitional care program; Empowering the person to self-manage the transitional care process and Empowering the Informal Caregiver.

**Conclusions:**

It is imperative to promote the coordination of transitional care, increase the functional capacity of the person and empower the informal caregiver.

**Supplementary Information:**

The online version contains supplementary material available at 10.1186/s12912-024-02050-4.

## Background

Population ageing, mostly due to an increase in average life expectancy and improved health care, has brought important social and longevity gains, but it has also increased the incidence of chronic and/or disabling diseases [1], and the consequent increase in the prevalence of chronic diseases [2]. Such illnesses lead to a loss of independence for the elderly, with 55% of them having severe functional impairment [3].

In line with the increase in the prevalence of chronic diseases, disabilities and dependency, complex challenges are emerging for health systems to ensure the quality and safety of care for citizens, especially for the most vulnerable. In recent years, changes in policies, organization and provision of care have taken this reality into account, particularly with the emergence of the National Continuing Care Network, in Portugal. However, the continuity of care between the hospital and the community and other available offers such as third sector institutions remains scarce, with some authors arguing that, for the elderly population, for example, the transition from hospital to home has a high risk of adverse events [4].

Continuity of care involves a complex set of interventions that are maintained over time, leading to improvements in the healthcare provided to the population and, obviously, their consequent satisfaction with the received care. Transitional care between the hospital and the community includes three distinct stages: before the person leaves the hospital; the moment of hospital discharge; and finally, the chronological arc between forty-eight hours and thirty days after discharge [5, 6].

The World Health Organization (WHO) highlights transitional care as a priority practice, which refers to moments of transition from hospital to home, home to hospital or even the transition in the health-disease situation that require a holistic and biopsychosocial approach to the transition of care, respecting the culture in which the person is inserted, involving the family/caregivers and community resources [7]. This multidimensional care implies the continuity of the relationship, information, management, and training of the informal caregiver (IC) and refers to an important body of knowledge about the organization and provision of care, implying advanced nursing practice [5–7].

The Transitional Care Model (TCM) was designed to prevent health complications and re-hospitalization of adults/elderly people with chronic illnesses, providing comprehensive discharge planning and follow-up care at home [8]. To this end, it advocates the identification of clients’ health goals, the planning and implementation of a simplified care program, as well as continuity of care between services and throughout the health-disease process. The provision of care, reflected in this model, should be monitored by the Advanced Nurse Practitioner, with the participation of the client, caregivers and the collaboration of members of the multidisciplinary team [7, 8].

The impact of this model of care is, according to information provided by the World Health Organization, very clear: with advanced practice nursing, it is possible to reduce hospital costs and readmission rates; home managed care, for example, provided identical or even more satisfactory outcomes when compared to the care provided to people who were admitted to hospital, reinforcing the reduction in costs, with an impact of around 17% [9].

There is a consensus that nurses play a fundamental role in identifying the needs felt by people with self-care difficulties as well as by their IC, as well as in appropriate choosing of interventions to ensure continuity of care between the hospital and the community. This role aims to minimize disabilities and promote knowledge and the acquisition of skills by the person and IC to adapt, often to the new health condition [9, 10].

To optimize the continuity of care between the hospital and the community, and to fully meet the needs of the dependent person, it is necessary to value and prioritize the needs of the IC. To achieve this, it is important to identify those needs and intervene so that the IC feels prepared to care not only for the dependent family member, but also for themselves. It’s important to note that stress, depression, and anxiety caused by providing daily care to the dependent elderly person can, by themselves, contribute to a deterioration in the IC’s wellbeing [7, 11].

The need to plan the transition of care, due to the complexity of dimensions associated with the provision of care as well as the consequent vulnerability of the hospitalization situation is a recurring theme in the professional’s discourse, who are aware that planning should be started as soon as possible to guarantee continuity of care [5, 7].

People who have been hospitalized are vulnerable to experiences of loss of this continuity, when there are changes in health status or when they move between care organizations [5, 7], having implications on their functionality and quality of life, as well as on the IC, translating into their health, the emotional, physical and social burden associated with the role of caregiver [7].

To this date, there are no known studies, in Portugal, that present the role of the specialist nurse in rehabilitation nursing in the organization and implementation of programs and interventions on hospital - home/community transitional care involving the person with dependency and/or rehabilitation nursing needs and informal caregiver.

Given that the problem stems from the way (rehabilitation) nursing care is organised, together with the international recommendations for advanced nursing nurses to lead the processes relating to continuity and integration of care, it is important to analyze the professionals’ perception of the phenomenon and gather contributes to ensure that transactional care is guaranteed safely and with health gains for this most vulnerable population.

In view of the above, the aim of this study is to analyze the perspective of the Specialists Nurse in Rehabilitation Nursing in relation to the organization and specialized intervention of transitional care for elderly people in need of rehabilitation programs.

## Method

### Study design

We chose a Qualitative Exploratory Study [12] to answer the research question: ‘What is the perspective of nurses specialists in rehabilitation nursing on the organization and specialized intervention of transitional care, to ensure the continuity of the rehabilitation programs, for older persons and their caregivers?”.

The choice of a qualitative study is justified because it makes it possible to work with complex phenomena, enabling in-depth study and bringing the experiences, knowledge and perspective of the participants to the solution of intricate questions of reality [13]. At the same time, the literature review revealed that there are few studies on the subject in Portugal, currently being only country in Europe with specialist nurses in rehabilitation nursing, limiting the comparison and dialog with similar experiences. For this reason, the study was carried out in three phases with the same objective.

In the first phase, a Focus Group (FG) took place, for which the protocol included the following stages: planning, preparation, moderation, data analysis and diffusion of results [14]. In a second phase, semi-structured interviews [12] were carried out with key informants for the purpose of the study. Finally, data was triangulated by combining it from different sources and collected using different techniques [15]. Triangulation differs from a combination of techniques, as it enables the interaction and analysis of findings obtained by each method and different techniques in order to analyze the same object of study [15, 16], leading to a greater understanding of reality and making it possible to unveil different aspects of the investigated phenomenon [16].

### Sample and recruitment

The eligibility criteria defined for the FG participants were: Nurses with Rehabilitation Nursing Specialty, involved in transitional care or in telemonitoring programs and/or telerehabilitation programs after hospital discharge, working in Portugal including its islands.

The eligibility criteria defined for the Qualitative Descriptive Study were: Nurses with Rehabilitation Nursing Specialty, who have implemented transitional care programs in the last 5 years, with process and outcome indicators for these programs.

The sample included nurses from hospital and primary care settings, all over the country, and was chosen to focus the discussion on the topic and deepen the results obtained in the FG.

Participants only participated in one of the studies, according to the pre-defined criteria.

### Data collection

Data was collected between April 2022 and February 2023. The FG took place in April, the findings were analyzed and then, the semi-structured interviews were conducted between October 2022 and February 2023.

For both studies, a semi-structured interview script was drawn up. The central question was kept and from there it was possible to ‘taper’ to more specific questions. Both central and secondary question, were intended to guide the interview, without conditioning the sharing. The interviews made it possible to gain a better understanding of the specific organization and specialized intervention of these nurses in relation to the transition of the person from hospital to the community and ensuring the continuity of care. The findings reached saturation in the 9th interview.

The FG was moderated by the study’s principal investigator, who also conducted the interviews. The FG was attended by a second researcher who took on the role of co-moderator [14]. Both the FG and the interviews were conducted online, using the Colibri® platform. The FG lasted around 2 h and 30 min and the interviews lasted on average, 65 min. The recorded data from the FG and the interviews were transcribed by the principal investigator.

### Data analysis

Following the recommendations of Carter et al. [17] when using both interviews and FG in a single study, the researcher must describe both methods of data collection, compare the study results from each method, and describe how the data were integrated to arrive at study results.

In the Focus Group analysis, the recommendations of Bloor et al. [18] were followed, to achieve a systematic and rigorous analysis, the findings were first coded by assigning categories and subcategories, then stored/retrieved, compiling and comparing all the text excepts under the same category, and finally, interpretation with systematic data analysis and respective inductive work. The process was carried out by two independent researchers using the WebQDA software®.

The interviews were also analyzed by two researchers independently, according to the content analysis technique [19, 20], following three stages: (1) Organizing the data; (2) Categorizing data by listing the central ideas and regrouping by similarity; and (3) Interpreting the data. The same qualitative data analysis software was used as in the previous study.

Subsequently, the results of the two studies were triangulated [17], the codes were compared to identify their differences and similarities, being sorted into main categories, each including subcategories.

### Study rigor

Relative neutrality and reasonable freedom from researcher bias with the following procedures [12] was ensured by following a protocol for each study, with a rigorous description of the procedures from planning to data collection, reporting personal assumptions and potential limitations.

The use of a QDAS (Qualitative Data Analysis Software) for data analysis not only allowed to assist the researcher in making large data sets more manageable but also to enhance the trustworthiness of the results [15].

On the other hand, the transcription of the interviews (FG and individual) and the repeated reading of them, allowed not only the immersion in the data, but also to have the sense of the whole [15], the constant comparison of the findings and the coding process between the researchers involved in codification, made it possible to guarantee the transparency of the inductive work. The interpretation made was validated by the team who have experience in conducting qualitative studies and was returned to the participants to validate the analysis and interpretation.

When defining the categories, objectivity, completeness, representativeness, homogeneity, exclusivity and relevance were ensured [19].

The methodological option of triangulation itself, has the potential to increase the validity of the study, decrease researcher bias, and add rigor, breadth, complexity and depth, by providing multiple perspectives of the phenomenon under study [15, 16].

### Ethical considerations

The research was authorized by an Ethics Committee (Protocol n.º 09/2019 HVFX). From the design of the study to the conduct of the investigation, ethical and legal principles relating to integrity, data protection, anonymity and confidentiality of the participants’ contributions were ensured. A code N (nurse) and a number (1,2.) were assigned to the Focus Group (FG) and Interviews (I).

The data is stored in an institutional cloud, protected by username and password, available only to two team members.

## Results

Eight nurses (five women and three men) took part in the FG. They work in different parts of the country, have worked for an average of twenty-seven years and have an average of twelve years of specialization (Rehabilitation Nursing).

Of the thirteen interview participants, twelve are women and have been working in Rehabilitation Nursing for an average of eight years.

The following categories emerged from the analysis: (1) Coordination of the transitional care program, with the subcategories team training, planning and continuity of individual rehabilitation programs and continuity of information between levels of care; (2) Training the person to self-manage the transitional care process, with the subcategories training for self-care, accountability and communication with the program manager; and (3) Training the Informal Caregiver with the subcategories transition to the role of caregiver and skills training and support to promote independence.

### 1) Coordination of transitional care program

Participants consider that nurses specializing in rehabilitation nursing are prepared for an advanced and differentiated approach that allows them to ensure the continuity of rehabilitation programs between the hospital and the community, specially because clinical discharge is accompanied by dependence on self-care and activities of daily living, which are central focuses of rehabilitation nursing care in Portugal. This alignment expresses that this is a priority area for people who need care, but also for the development of rehabilitation nursing care, as this nurse notes:

During this phase of COVID and now in the post-COVID period, we see that people are increasingly dependent, with complex needs and it’s not just older people, it’s everyone who has a chronic illness, disabilities or fewer resources and needs long-term care, many of them, rehabilitation and lifestyle changes, but these needs have had little response because the health systems are weakened after the pandemic, there is demotivation, lack of personnel and resources, it almost seems that the needs have increased and the capacity to respond has decreased (…) I think there is not only the need, but also the opportunity to organize ourselves to accompany these people and provide them with programs that allow them to live better, even with some limitations they may have. ” (IN5).

This configuration makes it imperative for teams to be trained in a philosophy of rehabilitation, with an increase in the participation of specialists in defining the professionals needed in the team, to ensure transitional care and for the training of other professionals in a work context, as expressed in these excerpts from the interviews:

“It is not a habit for us to discuss transitional care needs and work on this need with the team, especially rehabilitation programs. Oh! There are these moments of sharing knowledge, which usually goes from specialist nurses to general care nurses, when we identify that something is not correct in the inpatient care. For example, someone didn’t perform the uprising that day, and the specialist nurse usually goes, “Ah, but why wasn’t there an uprising? There is benefit, this has already been demonstrated.” more in the sense of understanding why the provision of care was that way and we tried to modify it, justifying what the best way could be, however we are not in the habit of doing this during shift changes or team meetings. This is an area that we need to improve both in terms of training and discussing cases among ourselves to prepare people for independence when they return to their homes.” (IN11).

“The presence of the social worker in team meetings and in the discussion of discharge situations was fundamental and that never happens and this is a huge failure, when I see that there is a need for support products or social support in the community, such as support from day center or home support, I always include the social worker in the referral of that patient and, therefore, what continued care is required. Basically, if it isn’t me after the meeting going to the social worker, “How are we, let’s see about this, family”. When the medical and nursing team meets, the social worker never joins [the meeting] and I would really like to change this.” (FGN3).

Experts make clear the need to gain space for team definition and training because the complexity of health situations, community resources and social needs that intersect with health needs imply a multidimensional response that is not limited to the scope of competences of a single professional group.

This multidisciplinary approach extends to the planning and continuity of individual rehabilitation programs, starting in the hospital and continuing upon return home. The analysis of nurses’ responses indicates that the Rehabilitation Program must include specific objectives and interventions for preparing for discharge and up to approximately 30 to 45 days after returning home, given that this is the critical period for readmissions and visits to the emergency room, with non-urgent situations, which could be resolved by community teams. It is consensual that it should begin during hospital admission, be individualized and centered on the person, with the total involvement of the person and caregiver in defining the interventions of the rehabilitation program, particularly those that are relevant to be maintained over time:

The passage, for example, of a patient from the hospital and then returning home, in a context where they still need care, will always allow them to be monitored and the last person to intervene will be able to assess whether there have been improvements or not. In other words, doing a cycle here, it’s fine, it stays in the community, there is a worsening, something goes back to the hospital, but preferably referred by the community team, often what brings them back are complications related to the absence of care nursing, such as problems associated with immobility syndrome, respiratory and urinary infections, dehydration. With due monitoring, these issues had been prevented in the community(…)” (IN2).

The program must have process and result indicators. The issue of indicators is made very clear in interviews with colleagues who already coordinate pioneering programs in this area of care, in Portugal, who recommend the definition in each context of a set of indicators that can allow the evaluation of the process and results in terms of outcomes for the client and health systems, focusing in particular on functionality after one, three, six and twelve months, number of emergency visits and readmission rate.

A central aspect to the success of the programs is the continuity of information between levels of care, as a nurse warns:

When they are discharged, give this knowledge so that these patients can be guided here. Knowledge must be given not only to the patient who has the possibility of continuity of care, but also to colleagues in primary healthcare (…), referrals, the vast majority of them, are a utopia, that is, we do not reach 1/3 or 1/5 of the users we should receive, due to lack of discharge notification. I worked at the hospital for years and never realized that this information does not reach health centers” (IN6).

When the person is discharged, they bring a discharge letter with information that is scarce in relation to the rehabilitation program, and which is often not delivered to primary health care, even due to the person’s lack of knowledge of the existence of rehabilitation nursing care in the community.

“The nursing notes are all the same, hence why a lot of information is missing. And I think we can start there. By writing more and individualizing more, and then if we have these new technologies, we could start exploring this aspect a lot more.” (FGN2).

There is a sub-optimization of existing resources due to non-notification of hospital discharge, which prevents not only the continuity of care, but also the management of other resources that exist in the community and that can help in the recovery of older people, such as technical aid banks, movement academies, municipal social support services and private social solidarity institutions. In this regard, colleagues who participated in individual interviews, perhaps due to their responsibility in implementing programs, warn for that transitional care:

“It will be a new aspect that rehabilitation nurses, particularly in home healthcare, will undoubtedly have to respond to. And above all, guarantee continuity and follow-up between the two levels of care. What my colleague said about her work in primary health care is very important, because people do need it, they need follow-up.” (IN13).

Furthermore, the articulation of information must incorporate new technologies, guaranteeing the protection of client data and promoting the dematerialization of discharge letters, allowing online access for both sick people and ICs as well as health professionals, who in community will ensure the continuity of the rehabilitation program. In both groups of participants, it is clear that the complexity of clinical situations combined with the housing characteristics of some people and the need for efficient management of scarce community resources is a challenge for new ways of articulating information, particularly the need for an assessment accessibility conditions and the presence of obstacles in the person’s home before returning home, especially in situations where that person was not yet followed by community teams, and/or due to the reason for hospitalization they became more dependent for self-care. A nurse observes the need to perform:

“(…) first of all, a multidisciplinary meeting with all colleagues who were involved with this user so that we could coordinate with the community. Second, make a pre-visit to this family to understand the concerns that exist in the community of that family and then be able to receive that user later. I think this should be ideal. From the assessment of needs, from the knowledge I have of that patient in the hospital to the problems I have at home, with family, I can then better receive and plan my care.” (FGN8).

In this category regarding the Coordination of transitional care programs, concerns are expressed regarding team training, planning and continuity of individual rehabilitation programs, continuity of information between levels of care to ensure safe and uninterrupted transitional care, centered on the person and family, taking into account personal, family and community resources, allowing access to local health care, assessing the need for changes in the home environment and support products to ensure self-care, promote independence and facilitate the continuity of the rehabilitation program. Furthermore, so that the coordination of the Transitional Care Program can do justice to coordination and vertical integration, it must provide opportunities for the use of technology, through tools and platforms, as well as the standardization of instruments for measurement of indicators relating to self-care, functionality and quality of life.

### 2) Train the person to self-manage the transitional care process

The specialists focus on empowering the person to self-manage the transitional care process based on their inclusion in planning the return home, continuity of care and reintegration into the community. It is inferred from the content analysis that the areas of action of the teams, embedded in a rehabilitation philosophy, are training for self-care and making the person responsible for this self-management and communication with the program manager.

With regard to training for self-care, they note that the continuity of the program not only allows for its progressiveness, with increased functional capacity, walking potential and increased independence in self-care, but also avoids what a nurse calls ‘syndrome of abstinence of the health technician’ (IN7), in the sense that leaving the hospital and the delay in being observed in the community and starting a new program means that, in many cases, the person spends a month without follow-up, abandoning the rehabilitation program started in the hospital, increased inactivity and decreased social participation, sometimes combined with family overprotection that leads to a vicious cycle of inactivity and dependence:

“Also because in the case of strength or walking training, it requires us to see if the person is doing the exercise correctly, it requires more visits. What we noticed is that they already walk around in the hospital with the help of the crutches, but when they get home, either out of fear or lack of space they stop doing so” (FGN5).

“Because they feel alone and abandoned when they get home, even managing the therapeutic regimen is difficult. All inhalation techniques are not used by them, they do not train, they have difficulties, and they cannot do it” (FGN1).

The specialists advocate that self-care training should be planned in the medium and long term and not just in the short term for the period of hospitalization because planning depends on:

“several things: the person’s condition, age, personality, even their social and family life, it involves many factors including what the home environment is like, whether there are stairs or not, whether or not it allows people to walk safely., the bathroom is safe, how are they already in family terms, with or without a family member who helps with self-care, to guide the caregiver and their needs.” (IE3).

“Regardless of this, for these users, the lack of knowledge also ends up being the fear and apprehension they have in facing these situations and, then, it is their family context and their home context, which is also extremely important, because they do not have resources.”(FGN4).

In terms of accountability and communication with the program manager, nurses are unanimous in considering that monitoring must start from the person’s needs and that they must be the ones to define what they need and how they communicate with the program manager, especially when this continuity is ensured. through new technologies, with telemonitoring and/or telerehabilitation. In these situations, the recommendation is that moments of care mediated by technology and moments of need for in-person monitoring should be defined with the person.

There is no consensus that technologies can increase a person’s responsibility. Some nurses warn that the use of technology does not, in itself, increase the health literacy of elderly people and for some it may be difficult to adapt to the interfaces used, which implies training for these types of care and making it clear that in this case of non-adaptation, the ‘traditional face-to-face modality is always possible.’ (FGN4).

Regardless of the option, or not, for different modalities of e-health, professionals recognize that the success of continuing the rehabilitation program and integrating transitional care depends on making the person and family responsible for communicating their needs and accessing professionals, clearly communicating difficulties, needs and giving feedback on self-perceived gains from the programs.

### 3) Training the informal caregiver

IC training emerges in the discourse essentially as a resource for continuity of care and not itself as a target of care in the face of the transitions it is experiencing.

“I think that family is very important and we often forget what they are experiencing. It’s just that the family is often worse off than the patient. I’ll give you an example, I have a man who took care of his grandson and, in the meantime, his grandmother and grandfather caught SARS-COVID-2 and the boy, who is bedridden, a child with muscular dystrophy, was the one who reacted best. So, the elderly man, who is very independent, goes to the hospital. When he arrives home in wheelchairs, which he wasn’t expecting, with changes in communication. How did this family react? In this situation, I didn’t know who I should help, whether it was the family or the patient himself. The truth is this, so we also have to prepare the family in which we are here and that we can. (FGN6)

It is clear that the concern is to promote their transition to the role of caregiver:

“in many situations the person was independent before going to the hospital and due to the consequences of the disease or associated complications, they are left with a tube, with gait changes and need help to return home, for hygiene care, for everything (…) and this is only possible if there is someone who can help, be it an informal or formal caregiver, it is increasingly difficult to train caregivers, family, to continue care, not only because many work, but also because they have never seen themselves in this paper, they have no idea what is needed, nor are they aware that it will not be for a short period because that person will need help for a long time (…) in a short period we have to make them understand that their family member needs of your help and at the same time prepare them.” (IN1).

In the professionals’ discourse, the nurse’s intervention to promote the transition to the role of caregiver is carried out simultaneously with the training of skills, which are considered central to ensuring the continuity of the program:

“We continue to have hospitalizations without visitors, which makes it impossible to train the caregiver, the family, the person who is closest; Even when there are visits, with reduced hospitalization times, it is very difficult to train them to ensure, or at least, supervise what the elderly have to do at home, in terms of activities and exercises.” (IN5).

Another concern closely linked to Portuguese culture and the way family ties are established and health-disease processes are experienced, is the support for promoting independence, avoiding overprotective behaviors motivated by illness and disability, which limit the elderly person’s autonomy and concomitantly increase dependence on self-care by replacing family in carrying it out.

“Families need to be prepared even in the way they should help their family member, not substituting, not creating dependence, whether because the elderly person is appealing and asks for help with everything, or because they are afraid that they will be encouraged to take a bath or eat alone, worsen the functional status. This is not an easy job, sometimes I see that the person has even been prepared in the hospital and knows what needs to be done and the elderly person knows that they have to continue getting out of bed, getting dressed, not to be in pajamas all day, but family relationships, emotional issues and other things that are not always clear to nurses, even the ones we come into the house every day and there is behavior that promotes dependency” (IN12).

IC training is central to ensuring the integration of transitional care and the continuity of rehabilitation programs initiated in the hospital. Training implies the acquisition of knowledge and the development of a set of instrumental skills for carrying out some care, as well as support to prevent overprotection and substitution behaviors.

## Discussion

Transitional care program coordination involves concerns about team training, planning and continuity of individual rehabilitation programs, and continuity of information between levels of care. In the speech of these participants, the need for team training to include a philosophy of rehabilitation and a multidimensional response, compatible with interdisciplinary work, is evident. Rehabilitation nurses, due to their specialized training in nursing, play a decisive role in the multidisciplinary team. They should be facilitating agents in transitional care, taking on the coordination of rehabilitation programs and projects, with health gains and a very positive influence on the quality of healthcare [21].

The nurse specializing in rehabilitation nursing, as a member of the team, must ensure transitional care, which is assumed to be health care extending to the hospital and with a community focus, aiming to prevent hospital readmission [6]. It includes the need to assess accessibility conditions and the presence of obstacles in the person’s home [9] before returning home, especially in situations where that person was not yet followed by community teams.

The planning and continuity of individual rehabilitation programs emerge in the findings regarding the coordination of transitional care programs. They must begin with hospital admission and continue during hospitalization and after discharge [6]. As hospital discharge is a specific transition with provision for continuity of care, its preparation requires the involvement of the person, their family members and the nursing team to ensure continuity of care, in a safe manner, avoiding re-hospitalizations [6]. The involvement of these actors involves a partnership of clients, informal caregivers and health professionals, working together, aiming to improve the experience and rehabilitation of the person, as object of their care [22].

Programs must be individualized and participant-centered, with the involvement and participation of patients and caregivers and adjusted to their capabilities. Educating the person and family for discharge and planning effective follow-up in the home context are fundamental to the success of transitional care [6]. Family participation in transitional care is crucial to the success and continuity of care in the home context. And, in this sense, the needs of IC/family caregivers must be identified and responded to for effective care. The knowledge, skills and tools necessary to participate in transitional care are a concern for nurses throughout this process [23].

All efforts are directed towards the transition of care, aiming to prevent the emergence of failures that could lead to the person being admitted to hospital. In the study carried out by Jones et al. [24], which aimed to understand the clients’ perspective on their care transition process, the conclusions point to the existence of multiple opportunities to improve processes, of which we highlight: providing systematic and reliable care transition processes for all, with follow-up telephone calls, numbers for sick people to call and delivery of medical equipment to the home, approach to social services to transport the person and, scheduling and helping the people involved to attend follow-up appointments. The strategies highlighted are essential to ensure adequate coordination and continuity of the programs to be developed with the participants [24].

Continuity of information between levels of care is crucial to the success of programs. Transitional care involves a set of measures to ensure that people receive continuous and safe and tailored health services to the person’s health situation, including discharge planning, monitoring and self-management of the care process during the period in which need to travel between the hospital and their home [25]. As it is a complex process and full of risks, there must be coordination in the provision of care, with effective communication in the transfer of information in relation to care and the rehabilitation program to be developed at home [1, 5, 25].

The use of information and communication technologies can be decisive in this process. Despite the electronic literacy problems of many older people, technology and interconnected electronic systems can be used as a resource to ensure continuity of information, care and safety of participants during the transition process [25, 26].

The standardization of instruments for measuring and indicators related to self-care, functionality and quality of life are part of an information continuity strategy [25, 27].

Training people to self-manage the transitional care process is one of the concerns of these nurses in the domain under study. It comprises the subcategories of training for self-care, accountability, and communication with the program manager. Still in a hospital context, the transition process to home requires training participants (people involved and caregivers) with information about medication and care adjusted to their health situation and a discharge letter that must be sent to primary health care. After discharge, the person and their family members will continue to be supported by nurses and other health professionals in the community to self-manage their condition [4].

Training for self-care is a challenge and a necessity, given that health problems worsen or interfere with performance in activities of daily living. It presupposes care directed towards the provision of care at home and home care that is consistent in its organization (scheduling) and in the construction of trusting relationships between participants, meaning client, family and home care providers [22]. Despite the importance of hospital care, it is necessary to optimize coordination and collaboration between the hospital and community sectors [22]. The home context must be valued, as it encourages participation in the co-production of health care, with a logic of coordination and planning of care, relational continuity, interprofessional collaboration and the possibility of adjusting to the person’s desire for direct care [22, 28].

To ensure the effectiveness of transitional care and to ensure continuity of information and training for self-care, it is important to use explanatory leaflets and follow-up after discharge, to guarantee the person’s motivation and adherence to the program [6, 29].

Person training is a process that must extend and consider home care. To this end, it is essential to understand care experiences from the client’s perspective. This implies improve communication between services and clients to identify barriers to access and continuity of care, as well as to identify factors that facilitate the quality of care and that can improve health services [22].

In the study carried out by Oikonomou et al. [4], six out of fifteen clients reported having experienced some problems in managing their care since returning home, such as delays in receiving health supplies such as catheters, the need for additional care that they did not expect or were not prepared for, or readmission to hospital.

The process of training participants in the development of programs involves making them responsible for the type of monitoring, which must be ensured throughout this process. The person must define the type of support they want in the development of the program, whether in person or using information and communication technologies. To make this entire training process viable, it is essential that there is adequate coordination in case management, as an integrated care strategy [30]. It is necessary that, associated with program planning, there is coordination and proactive monitoring of care, a relationship with the program manager and support for self-management to improve health outcomes and collaborative work between health and social professionals [31]. This process of accountability and coordination with the program manager is based on the assumption that the person’s perspective is the organizing principle of integrated care. And, this vision of person-centered and coordinated care allows participants to be involved in care planning, decision-making and the self-management process [31].

Caregiver training is highlighted by these participants as a resource for continuity of care. The nurse’s intervention focuses on promoting the transition to the role of caregiver and skills training, aiming at the continuity of the program. To this end, it is essential that nurses, during moments of involvement of family caregivers and in particular, when planning discharge, are able to adjust their vocabulary to the person’s intellectual and cultural level, and use clear, objective and effective communication to facilitate the understanding of information and the acquisition of knowledge and skills [6]. This process should motivate the person to actively assume the role of caregiver. This training process should lead you to understand the health status of the person being cared for, the procedures and routines and the entire care plan that must continue in the future care context, the home [6, 23].

Caregiver training presupposes that guidance and preparation by the nurse helps to organize the daily life of the person undergoing the rehabilitation process. To do this, the nurse must gather information about the clinical history, family network, social support networks, home conditions, medications, care program to be developed and appointment scheduling [6, 23]. Home visits to the person receiving care at home and their caregiver are essential to assess the impact of strategies implemented in the transition of care and to guide and support the caregiver. This work to train caregivers is part of the partnership work that must be established between the healthcare team, the person involved and family caregivers [6].

In the secondary study carried out by Ferreira et al. [5] on transitional care for caregivers of dependent elderly people, made it possible to highlight five categories of transitional care needs for caregivers, compatible with the management of care for the caregiver themselves:

- needs in the transition to the role of caregiver, related to information needs, care skills and emotional support and prevention of overload;
- his own self-care needs, related to reconciling the new role with the management of family dynamics, other roles of the person and social relationships;
- health needs, related to the health-disease processes experienced by caregivers, namely situations that can lead to physical and/or mental illness;
- economic needs, associated with financial expenses to obtain material and human resources for the care of dependent elderly people;
- social and community needs, related to transport assistance, special equipment to respond to the physical needs of the dependent elderly person, the caregiver’s need for rest and home support services that assist in providing care at home, especially in situations of greater vulnerability [5].

In this line of thought, in the study by Alvarez et al. [3] using a descriptive approach, it was found that the learning needs among family caregivers focused more on understanding medical conditions, drug administration, care for the elderly, diet and nutrition, walking strategies, support financial and total management of care for elderly people. Family caregivers were valued for their knowledge, skills, attitudes and values regarding the care of elderly people. Satisfying these learning needs based on knowledge, skills, attitudes and values has an impact on the health of family caregivers, preventing them from experiencing situations of despair, depression and self-isolation [3].

Support and training for informal caregivers of dependent people was the object of study of a nursing intervention project developed by Melo et al. [1]. In the first stage, an integrative literature review was carried out, which allowed the main caregiver needs to be identified. Afterwards, a nursing intervention program was implemented, focusing on the domains of emotional and instrumental support. This program was validated by experts, using the Delphi technique. Its implementation was carried out using a quasi-experimental study, with pre and post intervention evaluation and as a result of this, there was a general improvement in the IC’s health status, with a reduction in manifested overload and a greater use of strategies of coping. The study showed that these nursing intervention strategies aimed at ICs facilitated the transition to the role of caregivers and had an impact on their health and the care provided to the dependent person [1].

### Implications for practice

We believe that the results of this study can be an important contribution to the reflection on this issue of transitional care and the relevance of specialized Rehabilitation Nursing intervention in this care reality.

Furthermore, these findings could make important contributions to the definition of health policies that improve the transition of care and IC training programs.

### Limitations of this study

The choice of a qualitative study carried limits the transferability of findings. Although it is a study that explores the perspective, there are aspects related to the professional and personal experience of the participants that may have influenced their response. Although the definition of inclusion criteria for the participants, it´s possible that may not have captured all perceptions about this topic.

The choice of a semi-structured interview script, which allows for some flexibility and increases the richness and depth of the findings, increases the difficulty in conducting it and the diversity of responses. Also the interaction between researcher and participant may have influenced the response to what is socially accepted.

Despite the limitation implied by it, we believe in its importance in order to increase the richness of the findings. The triangulation of methodologies led to a greater understanding of the reality surrounding transitional care, increasing the rigor, depth, extension and the transferability of findings that can only be perceived in light of this reality and the cultural specificities of the participants. Data analysis involved the participation of experts, with expertise in the topic under study and whose intervention was decisive in the analysis of the coding and categorization process. On the other hand, the results of the analysis were returned to the participants to validate the main researcher’s interpretations, in addition to the possible concern with the explanation of the data collection and analysis procedures, as well as the theoretical perspective that framed the study. An enormous effort was made to try to overcome possible limitations and to safeguard the rigor that is required for a study of this nature.

## Conclusion

This study allowed us to understand the organization and specialized intervention of transitional care for elderly people in need of rehabilitation programs, from the perspective of rehabilitation nurses.

It was possible to characterize how transitional care is organized and how people and their caregivers are trained. The positive aspects and difficulties encountered in each dimension were presented and strategies were identified to minimize them. In summary, the coordination of transitional care must be promoted to increase people’s functional capacity and also train the IC, ensuring safe care, in order to reduce the appearance of post-discharge complications, and make health systems more sustainable, avoiding readmissions and premature deaths.

### Electronic supplementary material

Below is the link to the electronic supplementary material.

Supplementary Material 1

## Data Availability

The data used during this study are available from the corresponding author, under request by e-mail.
