# Supplementary material for: Transitional care for older persons with need of geriatric rehabilitation nursing interventions
Source: BMC Nurs. 2024 Jun 4;23:376. doi: 10.1186/s12912-024-02050-4 (PMC11149285; doi:10.1186/s12912-024-02050-4)
Supplement: Supplementary file 1 — Supplementary Material 1 [file 12912_2024_2050_MOESM1_ESM.docx]

**Semi-structured interview for Focus Group and individual interviews**

**Main question**

What is your perspective on the organization and specialized intervention of transitional care, for the elderly with rehabilitation programs maintained over time?

**Subsidiary/Secondary questions**

1 - In your opinion, what are the main difficulties in guaranteeing continuity of rehabilitation care between the hospital and the community? (What factors contribute to this?)

2 - During hospitalization, what are the limitations in preparing to return home (with a focus on specialized care from rehabilitation nurses)?

3 - What do you see as the difficulties in preparing for discharge?

4 - In the first 2/3 days after returning home, what are the obstacles/difficulties to continuing the care/rehabilitation programmes started in hospital?

5 - From your perspective, how do these difficulties impact on continuity of care in the 1st month to 45 days after discharge?

6 - What strategies should be implemented, by rehabilitation nurses, to ensure the transitional care?
